# Ultrafast response of harmonic modelocked THz lasers

**DOI:** 10.1038/s41377-020-0288-x

**Published:** 2020-04-01

**Authors:** Feihu Wang, Valentino Pistore, Michael Riesch, Hanond Nong, Pierre-Baptiste Vigneron, Raffaele Colombelli, Olivier Parillaud, Juliette Mangeney, Jerome Tignon, Christian Jirauschek, Sukhdeep S. Dhillon

**Affiliations:** 1 Laboratoire de Physique de l’Ecole Normale Supérieure, ENS, Université PSL, CNRS, Sorbonne Université, Université de Paris, Paris, France; 2 Department of Electrical and Computer Engineering, Technical University of Munich, Arcisstr. 21, 80333 Munich, Germany; 3 Centre de Nanosciences et de Nanotechnologies, CNRS, Univ. Paris-Sud, Université Paris-Saclay, C2N-Orsay, 91405 Orsay, Cedex France; 4 III-V Lab, 1 avenue Augustin Fresnel, 91767 Palaiseau, France

**Keywords:** Quantum cascade lasers, Photonic devices

## Abstract

The use of fundamental modelocking to generate short terahertz (THz) pulses and THz frequency combs from semiconductor lasers has become a routine affair, using quantum cascade lasers (QCLs) as a gain medium. However, unlike classic laser diodes, no demonstrations of harmonic modelocking, active or passive, have been shown in THz QCLs, where multiple pulses per round trip are generated when the laser is modulated at the harmonics of the cavity’s fundamental round-trip frequency. Here, using time-resolved THz techniques, we show for the first time harmonic injection and mode-locking in which THz QCLs are modulated at the harmonics of the round-trip frequency. We demonstrate the generation of the harmonic electrical beatnote within a QCL, its injection locking to an active modulation and its direct translation to harmonic pulse generation using the unique ultrafast nature of our approach. Finally, we show indications of self-starting harmonic emission, i.e., without external modulation, where the QCL operates exclusively on a harmonic (up to its 15th harmonic) of the round-trip frequency. This behaviour is supported by time-resolved simulations of induced gain and loss in the system and shows the importance of the electronic, as well as photonic, nature of QCLs. These results open up the prospect of passive harmonic modelocking and THz pulse generation, as well as the generation of low-noise microwave generation in the hundreds of GHz region.

## Harmonic help boosts Terahertz laser

Ultrafast terahertz and sub-terahertz communications could be enabled by a new infrared laser developed by researchers in France and Germany. Sukhdeep Dhillon at the Ecole Normale Superieure in Paris and co-workers built a quantum cascade laser that emits rapid pulses of far-infrared radiation, close to the microwave region. Their laser undergoes so-called harmonic mode locking, meaning that ultra-short pulses are generated, separated by intervals even shorter than the time it takes a photon to do a round trip of the laser resonator cavity. The researchers observed very stable mode locking at the second harmonic, with hints of other frequencies up to a 15th harmonic, enabling the laser to emit pulses at previously unseen rates. This was a result of considering the electronic as well as photonic properties of the laser, which operates at the frontier between these methods to generate electromagnetic radiation. As well as enabling high-bit-rate data transfer, such lasers could detect gases or probe ultrafast dynamics and phenomena across the physical, chemical and biological sciences.

## Introduction

Harmonic modelocking is routinely used in the visible and near-infrared range and consists in the generation of multiple light pulses within the photon round-trip time of a laser cavity. This offers the possibility of high-repetition-rate laser systems, reaching GHz and tens of GHz rates, beyond rates that are limited by the laser cavity length. This is of particular interest in high-bit-rate optical communication^1^, photonic analogue-to-digital conversion^2^, multi-photon imaging^3^, and astronomical frequency comb generation^4^. Furthermore, modelocked lasers with high repetition rates can be applied to microwave photonics for radio frequency arbitrary waveform synthesis and for the generation of extremely low-noise, high-frequency sub-mm waves in future wireless networking technologies^5^. However, no demonstrations of harmonic modelocking have been shown in the terahertz (THz) range with quantum cascade lasers (QCLs)^6,7^, which are one of the only practical THz semiconductor lasers^8^. Unlike standard interband lasers, harmonic active modelocking is inherently adapted to QCLs, as the unique fast dynamics with picosecond relaxation dynamics permit an ultrafast modulation of the gain and loss. Multiple demonstrations of fundamental active modelocking in QCLs have been reported, where the QCL is electrically modulated at or close to the round-trip frequency^9–11^ (f_RT_ = *c/*2*nL*, where *n* is the refractive index of the material, *c* is the speed of light in vacuum and *L* is the cavity length, assuming no refractive index dispersion). Recently, MIR QCLs have shown self-starting harmonic modelocking^12^, and indications were observed in the THz range^13^, but no measurements of the ultrafast origins or time behaviour have been shown thus far.

**Fig. 1:**
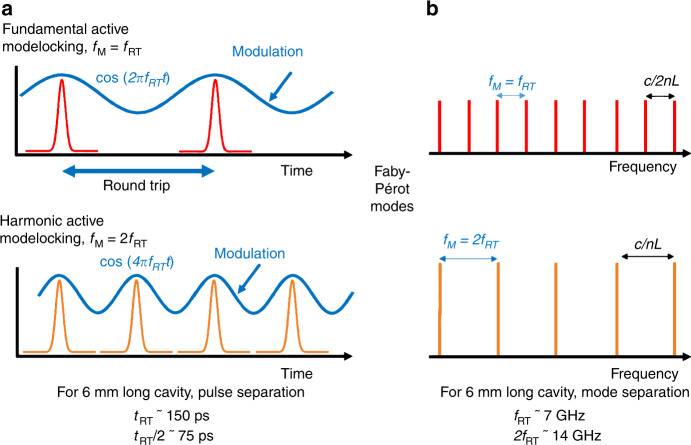
Fundamental and harmonic modelocking in the a time and b frequency domains. Fundamental modelocking: In the time domain, modelocking corresponds to an active modulation that is synchronised to the round-trip time of the pulses. In the frequency domain, this corresponds to a modulation that is equal to the mode spacing, i.e., the round-trip frequency (f_RT_). Harmonic modelocking: In the time domain, second harmonic modelocking corresponds to an active modulation that is half the round-trip time of the pulses. In the frequency domain, this corresponds to a modulation that is twice the round-trip frequency

In this paper, we show the first demonstrations of harmonic active modelocking of QCLs, as well as self-harmonic emission at multiple (15th) harmonics with 15 pulses per round-trip. Importantly, we directly measure the time response and show that the latter has its origins in the ultrafast gain dynamics of THz QCLs that permit high-frequency microwave generation, which in turn modulates the gain and loss of the system. These effects are unique to THz QCLs, showing both electronic and photonic behaviour. This work further highlights control of the spectral mode spacing at harmonics of the round-trip frequency and pulse generation at multiples of the cavity length, resulting in greater than one pulse per round-trip. Figure 1 shows the concept of fundamental and harmonic modelocking in the time domain, showing a microwave modulation at the fundamental and second harmonic frequencies. For fundamental modelocking, the gain is modulated at f_RT_, resulting in pulses separated by the cavity round-trip time, with no pulses in-between. However, in the case of the higher-order (second) harmonic modelocking, the gain is modulated at 2f_RT_, providing for inter-round trip pulse generation. This results in two pulses per round-trip and an improved use of the QCL gain, permitting a higher signal-to-noise ratio for a given time window. For example, for a 6 mm long cavity, this would result in pulses separated by 140 ps (round-trip time) and 70 ps for the fundamental and the second harmonic frequency, respectively. Equivalently, harmonic modelocking can also be schematized in the frequency domain. (i) In the case of fundamental modelocking, sidebands are generated at or close to f_RT_. (ii) For harmonic modelocking, sidebands are generated at twice the Fabry-Pérot mode spacing, resulting in a spectrum with a mode spacing of 2f_RT_. For the example of a 6 mm cavity, this would correspond to a mode spacing of ~7 GHz and 14 GHz for the fundamental and second harmonic modelocking. (Note that a previous work has shown multiple pulses per round trip in THz QCLs^14^. However, this observation was based on modulation at the fundamental frequency, i.e., modulation at f_RT_.)

## Results

In this study, QCLs with a centre lasing frequency of ~2.5 THz with an emission bandwidth of up to 400 GHz are investigated based on a scaled hybrid active region operating at 3.2 THz^15^. (Details of the growth are available in the Materials and methods section.) This particular design was used to obtain a low current threshold for laser action, permitting the realisation of long cavities and highlighting the effect of the self-generated harmonic modelocked state. The wafer was processed into metal–metal (MM) waveguides using standard lithography, with the ridge defined using ICP etching for a vertical ridge profile. The ridge width was 60 µm, limiting higher-order modes, and the total length of the cavity was 5.9 mm, with the sample mounted on a high-speed mount (cut-off frequency >18 GHz). The Light-Current-Voltage (LIV) characteristics of the sample is shown in the supplementary section.

**Fig. 2:**
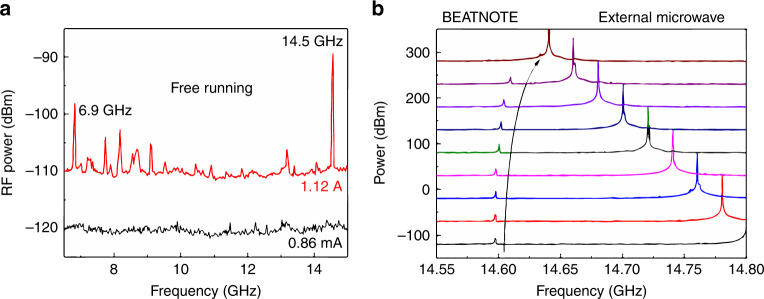
Beatnote and injection locking at the harmonic frequency. **a** RF spectrum collected with the QCL on and no RF injection, showing strong beatnotes at the fundamental (6.9GHz) and second harmonic (14.5GHz) round-trip frequencies. **b** RF injection locking of the second harmonic beatnote with the external microwave modulation (power: 16dBm) brought closer in frequency to that of the beatnote (The spectra have been offset for clarity.)

Prior to pulse demonstrations, initial measurements were conducted to investigate the electrical beatnote measured^16^ on the QCL up to 18 GHz, limited by the spectrum analyser. This method permits the observation of the beating of the Fabry-Pérot modes and determination of their frequency spacing and is often used to illustrate frequency comb operation^17,18^. Figure 2a shows an example of the spectrum at 1.12 and 0.86 A (above and below laser threshold, respectively). The strongest electrical beatnote is observed at 14.5 GHz (−88 dBm), corresponding to the second harmonic round-trip frequency, which has not been previously reported. Importantly, the second harmonic beatnote is more intense in power than any other feature, illustrating that the QCL tends to emit a spectrum with a mode spacing at the second harmonic, which is discussed further below. Smaller and wider beatnotes are observed below the harmonic; these beatnotes are possibly a result of interactions between different lobes of the spectral emission, as discussed in Li et al.^13^, or the presence of irregular modes in the spectrum (see below on the free-running harmonic properties and spectrum of QCLs). The feature at 6.9 GHz does not correspond to the fundamental frequency, as it is present only at this current, and no regular mode spacing at the fundamental frequency is observed in the spectrum. An important precursor to demonstrating active modelocking is to show that the measured electrical beatnote can be locked to an external microwave reference, i.e., radio frequency (RF) injection locking. Although extensively shown for the fundamental harmonic^16^, no demonstrations have been realised on a harmonic; this is shown in Fig. 2b for a QCL current of 987 mA and a measured beatnote at 14.6 GHz. Here, a microwave modulation with a power of 16 dBm is applied and gradually brought closer in frequency to the QCL beatnote. The latter is ‘pulled’ with a nonlinear dependence and eventually becomes locked to the microwave modulation. This pulling effect and the subsequent injection locking indicate that the Fabry–Pérot modes of the laser are mutually phase-locked to the microwave modulation. Here, we show that locking can be performed on a harmonic of the round-trip frequency, therefore operating in a regime of harmonic active modelocking.

**Fig. 3:**
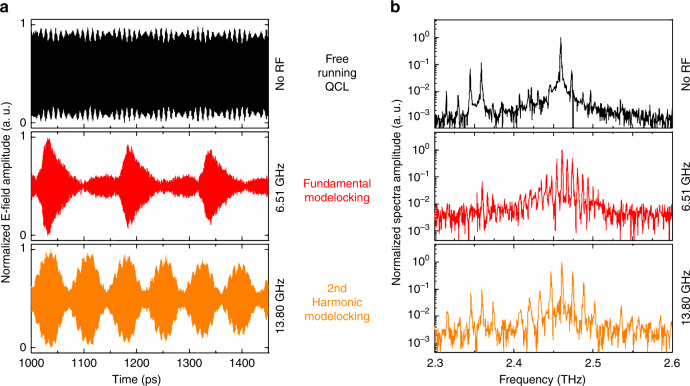
Comparison in the time and frequency domains of free-running (black), fundamental (red) and harmonic (orange) modelocking. **a** Time traces for a free-running QCL showing quasi-CW behaviour, fundamental modelocking with 1 pulse per round-trip and second harmonic modelocking with two pulses per round-trip. **b** Spectrum showing a limited number of modes for the free-running QCLs, with an enhanced number of modes for the fundamental and second harmonic modelocked QCLs with a mode separation equal to the round-trip and twice the round-trip frequency, respectively

The temporal profile of modelocked or frequency comb QCLs operating on the fundamental frequency has very recently garnered considerable attention, with a variety of new techniques applied, ranging from SWIFT^19^, phase stablization^20,21^ to intensity autocorrelations^22^. The time and spectral characteristics of the sample were investigated in this work using the established technique of injection seeding, which permits the amplitude and phase of the QCL emission to be determined on femtosecond time scales, from the build-up of the electric field to steady-state laser action (see Materials and methods section)^10,11,23^. Although this technique requires a seed pulse, the time profile when the QCL is phase-locked is equivalent to the QCL emission initiated by its spontaneous emission but coherently resolved with femtosecond resolution^24^. Figure 3 compares the ultrafast time profiles (left) and the corresponding spectrum (right, through a Fourier transform) for three different cases with the laser just above the threshold: (i) free-running QCL (black), where no active microwave modulation is applied; (ii) active modelocking at the fundamental harmonic at 6.5 GHz (red), and (iii) active modelocking at the second harmonic at 13.8 GHz (orange). The microwave power applied for modelocking was 18 dBm. In the case of the free-running QCL, a quasi-CW emission profile is observed owing to a predominately single mode operation at 2.46 THz in the frequency domain. A small modulation in the time profile is observed owing to another emission lobe at 2.35 THz and is further discussed below.

The situation changes drastically with an active microwave modulation. As in previous demonstrations, modulation at the fundamental round trip permits the generation of pulses separated by the round-trip time (~150 ps), resulting in more modes being brought above threshold with a Gaussian distribution and a mode separation at the fundamental round-trip frequency (6.5 GHz). Where this work differs from previous investigations is the modulation at the second harmonic. By modulating at 13.8 GHz, two pulses per round-trip are observed, with an extra pulse observed in-between the round trips, resulting in pulses separated by ~70 ps. (The drop in the electric field with time is a result of a non-flat RF modulation used to measure the QCL electric field profile.) The effect of two pulses per round trip is also clearly seen in the spectrum, again illustrating a Gaussian distribution but with double the mode separation of the Fabry–Pérot modes compared with the fundamental modelocking case. It can also be observed that the signal-to-noise ratio in the spectrum is higher for the harmonic modelocked case owing to the concentration of the field in fewer modes.

Compared with previous demonstrations where pulses down to 4 ps have been measured^11^, the pulses here are long, with 25 and 34 ps for the fundamental and second harmonic, respectively. There are two possible reasons for this result: the modelocked spectral bandwidth is limited because no dispersion compensation has been applied, and the cavity is long, resulting in a larger group delay dispersion (GDD). Shorter pulses can be realised via dispersion compensation with, for example, an integrated Gires–Tournois interferometer^11^.

**Fig. 4:**
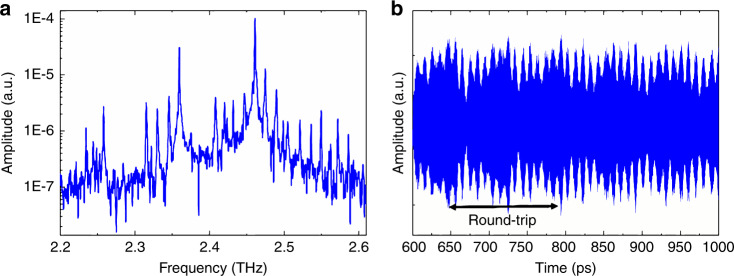
Self-starting high-harmonic emission. **a** Free-running QCL spectrum showing several frequency bands separated by 100GHz corresponding to the 15th harmonic of the round-trip frequency. Each frequency band has a mode spacing corresponding to the second harmonic of the round-trip frequency. **b** Time-resolved electric field profiles with multiple pulses per round trip and an ‘over-modulation’ at half the round-trip time

The final part of this paper reports the time-resolved natural operation of the QCL at a harmonic frequency and the potential indications of harmonic modelocking without an active modulation, i.e., self-starting. The latter has recently been reported for MIR^12^, as well as indications in THz QCLs^14^ but without direct measurements of the time profile. The first indication that the THz QCL here prefers to operate at the harmonic is the observation of the strong electrical beatnote at the harmonic frequency, which also suggests a mode coherence and equal mode spacing between spectral lines (see Fig. 2). Figure 4a, b shows the free-running (i.e., no external modulation) spectrum and field, respectively, at a slightly higher current than in Fig. 3, to bring further modes above threshold. In Fig. 4a, the spectrum shows a distribution of modes over 800 GHz with different spectral ‘bands’ at 2.25, 2.35 and 2.45 GHz. Notably, each of these bands shows a mode separation of 2f_RT_, i.e., already at the second harmonic. The time behaviour is shown in Fig. 4b, which illustrates a multi-pulse behaviour between round trips (~150 ps). The multi-pulses are separated by ~10 ps, corresponding to a frequency of 100 GHz and therefore giving rise to the different bands observed in the spectrum in Fig. 4a. This indicates the generation of the 15th harmonic and therefore potentially an electrical beatnote at ~100 GHz. (Unfortunately, this is beyond the range of our current spectrum analyser.) This fast modulation is linked to the gain recovery time^12^, which is on the same order as the oscillations and discussed below. The time profile is complex, but a clear modulation of the amplitude is observed, resulting in multiple ‘pulses’ per round-trip. It is also clear that the behaviour is not random and repeated for every half round-trip profile of the emission, which is in direct correspondence to the mode separation of 2f_RT_. This second amplitude modulation between round trips highlights that the QCL, even without an active modulation, can operate at a higher harmonic. In the case of self-locking in the MIR, it was proposed that the suppression of adjacent cavity modes originates from a parametric contribution to the gain, owing to temporal modulation of the population inversion. This exhibited a mode spacing up to the 50th harmonic, owing to the shorter gain recovery times in MIR QCLs. Further work in the MIR also showed the presence of a population inversion grating within the cavity^25^, illustrating the generation of second harmonic microwave radiation. In our work, operation at the second harmonic is privileged compared with the first, and this permits the generation of an intense beatnote at 2f_RT_ and a spectrum with a mode separation of 2f_RT_^26^. An important point is that the QCL here naturally operates at the second harmonic, most likely owing to the fact that this QCL has low electrical power requirements combined with a broad spectral response compared with previous studies^10,27^, exalting the effect of the self-generated microwave signal here.

## Discussion

**Fig. 5:**
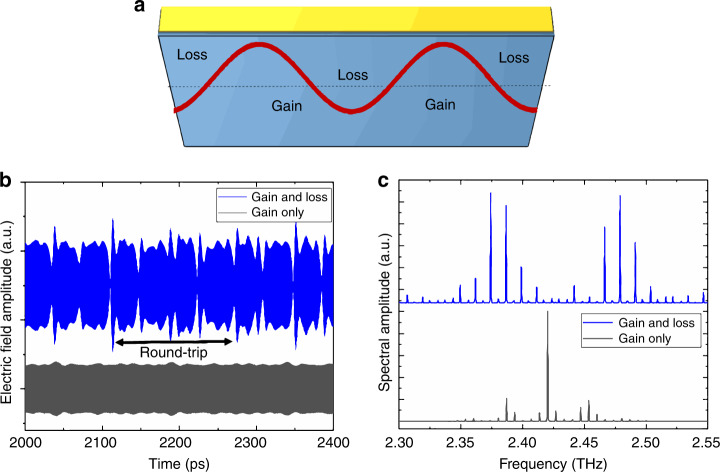
Simulated frequency response of a THz QCL using the Maxwell-Bloch formalism. **a** Schematic of a self-generated microwave signal within a QCL cavity that modulates the gain, resulting in interlaced loss and gain regions in the cavity. **b** Simulated response with constant gain (without loss regions), resulting in a mode spacing at the fundamental frequency. **c** Simulated response with loss regions, resulting in a mode spacing at the second harmonic frequency and frequency bands separated by 100GHz

To demonstrate the effect of the harmonic beatnote on the modulation of the QCL, numerical simulations based on the open-source software package *mbsolve*^28,29^ were realised to investigate the electric field evolution in a structure reproducing the QCL characteristics. The simulation model is based on the full-wave Maxwell–Bloch (MB) equations^30,31^ for the three-level system (i.e., without the rotating wave and slowly varying amplitude approximations), taking into account counter-propagating waves and spatial hole burning. Further details about the specific parameters used for the simulations as well as the time response can be found in the supplementary section. The simulations of the spectral response were realised in two cases: (i) the harmonic beatnote introduces loss regions along the structure and (ii) where the active region is above the threshold as a whole (i.e., no modulation). Figure 5a shows the optimal scheme that was considered, where the beating of the Fabry–Pérot modes generates a harmonic microwave modulation giving rise to alternating absorption (A) and gain (G) regions. Here, we have chosen a scheme that consists of a A-G-A-G-A set-up. As Fig. 5 of the spectrum indicates, the presence of the loss regions is an important condition to successfully reproduce the mode distribution separated by 2f_RT_ and the different spectral bands that were experimentally observed. Without the loss regions, the QCL operates on the fundamental round-trip frequency and shows no strong modulation of the electric field as a function of time. This suggests that the self-modulation is a potential source of harmonic emission in THz QCLs. Of note is that if the QCL consists of G-A-G regions, the QCL will also operate on the harmonic mode spacing, but the multiple pulses per round trip and the different spectral bands separated by 100 GHz are not reproduced (see the supplementary materials).

As mentioned above, the observation of a harmonic electrical beatnote suggests a mode coherence, indicating that the QCL is by itself operating in a self-modelocked harmonic state. However, the optical beatnote would need to be detected to further explore this behaviour. Furthermore, these observations of a higher-order electrical beatnote suggest the presence of a second-order nonlinearity that could assist the stabilisation of the QCL emission, as observed in MIR QCLs^32^. QCL combs have previously been believed to be stabilised by solely a third-order nonlinearity. This presence of a second-order nonlinearity would open up the possibility of the generation of hundreds of GHz emission from THz QCLs through difference frequency generation, which will be limited only by the spectral bandwidth of THz QCLs that can reach an octave^18^.

To conclude, our experimental results show harmonic modelocking of THz QCLs, where QCLs can be actively modelocked at the second harmonic of the cavity round-trip frequency. This process permits the generation of two pulses per round trip and a mode separation of twice the round-trip frequency. This potentially will allow an increase in the signal-to-noise ratio of QCL frequency combs (FCs) owing to the larger number of pulses per unit time. Furthermore, indications that the QCL can operate spontaneously (without external modulation) on the second harmonic are presented, which potentially further stabilises the QCL emission at the second harmonic frequency. Our realisation of second-order harmonic modelocking paves the way for higher-order (third, fourth, fifth, etc.) harmonic modelocking, taking advantage of the fast QCL dynamics, for high-repetition-rate applications. Observations of harmonic mode spacing at 15f_RT_ are also demonstrated, opening up the possibility of hundreds of GHz generation from low-noise modelocked QCL sources^33^.

## Materials and methods

A QCL with a ‘hybrid’ scheme was used, based on a 3.1 THz QCL^15^. The design was modified to operate at lower frequencies (~2.45 THz) by increasing the well and barrier widths. Starting from the injection barrier, the well and barrier widths were *4.5*/10.3/*4.1*/12.7/*1.9*/12.1/*5.5*/19.8 nm (Al_0.15_Ga_0.85_As barriers in italics). The 19.8 nm wide well was n-doped at a level of 2 × 10^16^ cm^−3^. The growth was performed using molecular beam epitaxy, and the wafer was processed into ridge waveguides using standard photolithography.

The pulse characterisation of the THz quantum cascade laser (QCL) is based on coherent sampling of the electric field (E-field) using electro-optic detection. This technique requires phase-locking the emission of the THz QCL to a THz pulse, which in turn is locked to the repetition rate of a femtosecond laser. To fulfil this requirement, an established ultrafast injection seeding technique is employed. A broad-band THz pulse (seed) with a fixed phase is generated using a photoconductive switch excited by a femtosecond Ti:Sapphire laser^34^. The THz seed pulse is injected into one end of the QCL waveguide prior to gain switching the QCL with an electrical radio frequency (RF) pulse with a duration of a few nanoseconds. This procedure allows the THz input pulse to be amplified and eventually seed the QCL emission instead of being initiated by the QCL’s inherent spontaneous emission. To initiate the modelocking regime, a microwave modulation of the QCL bias is applied close to the THz cavity round-trip frequency. The microwave modulation is generated from the photo-excitation of an ultrafast photodiode by a pick-off beam of the Ti:Sapphire laser. The generated electrical signal consists of a comb of frequencies extending to ~20 GHz separated by the Ti:Sapphire repetition rate (76 MHz). An yttrium iron garnet bandpass filter is used to pick out a harmonic of the reference laser repetition rate close to the QCL cavity round-trip frequency, which is then amplified by a set of microwave power amplifiers.

## Supplementary information

Supplementary Material

## Data Availability

The datasets generated and analysed during the current study are available in the zenodo repository, 10.5281/zenodo.3689614.

## Code availability

The simulation source code is publicly available under the terms of the GNU General Public License v3.0 in the GitHub repository^28^. The simulation software version v0.3.0 was used during this study. This version is available in the zenodo repository, 10.5281/zenodo.3694288.

## References

[1] Keller U (2003). Recent developments in compact ultrafast lasers. Nature.

[2] Villanueva GE, Ferri M, Perez-Millan P (2012). Active and passive mode-locked fiber lasers for high-speed high-resolution photonic analog-to-digital conversion. IEEE J. Quantum Electron..

[3] Voigt FF (2017). Multiphoton in vivo imaging with a femtosecond semiconductor disk laser. Biomed. Opt. Express.

[4] McFerran JJ (2009). Échelle spectrograph calibration with a frequency comb based on a harmonically mode-locked fiber laser: a proposal. Appl. Opt..

[5] Fortier TM (2011). Generation of ultrastable microwaves via optical frequency division. Nat. Photonics.

[6] Faist J (1994). Quantum cascade laser. Science.

[7] Köhler R (2002). Terahertz semiconductor-heterostructure laser. Nature.

[8] Williams BS (2007). Terahertz quantum-cascade lasers. Nat. Photonics.

[9] Barbieri S (2011). Coherent sampling of active mode-locked terahertz quantum cascade lasers and frequency synthesis. Nat. Photonics.

[10] Freeman JR (2013). Electric field sampling of modelocked pulses from a quantum cascade laser. Opt. Express.

[11] Wang F (2017). Short terahertz pulse generation from a dispersion compensated modelocked semiconductor laser. Laser Photonics Rev..

[12] Kazakov D (2017). Self-starting harmonic frequency comb generation in a quantum cascade laser. Nat. Photonics.

[13] Li, H. et al. Dynamics of ultra-broadband terahertz quantum cascade lasers for comb operation. *Opt. Express* **25**, 33270–33294 (2015). 10.1364/OE.23.033270 26831993

[14] Mottaghizadeh A (2017). 5-ps-long terahertz pulses from an active-mode-locked quantum cascade laser. Optica.

[15] Amanti MI (2009). Bound-to-continuum terahertz quantum cascade laser with a single-quantum-well phonon extraction/injection stage. N. J. Phys..

[16] Gellie P (2010). Injection-locking of terahertz quantum cascade lasers up to 35 GHz using RF amplitude modulation. Opt. Express.

[17] Burghoff D (2014). Terahertz laser frequency combs. Nat. Photonics.

[18] Rösch M, Scalari G, Beck M, Faist J (2015). Octave-spanning semiconductor laser. Nat. Photonics.

[19] Burghoff D (2015). Evaluating the coherence and time-domain profile of quantum cascade laser frequency combs. Opt. Express.

[20] Cappelli F (2019). Retrieval of phase relation and emission profile of quantum cascade laser frequency combs. Nat. Photonics.

[21] Consolino L (2019). Fully phase-stabilized quantum cascade laser frequency comb. Nat. Commun..

[22] Benea-Chelmus I-C, Rösch M, Scalari G, Beck M, Faist J (2017). Intensity autocorrelation measurements of frequency combs in the terahertz range. Phys. Rev. A.

[23] Oustinov D (2010). Phase seeding of a terahertz quantum cascade laser. Nat. Commun..

[24] Freeman, J. R. et al. Laser-seeding dynamics with few-cycle pulses: Maxwell-Bloch finite-difference time-domain simulations of terahertz quantum cascade lasers. *Phys. Rev. A* **87**, 063817 (2013).

[25] Piccardo M (2018). Time-dependent population inversion gratings in laser frequency combs. Optica.

[26] Tzenov P (2018). Passive and hybrid mode locking in multi-section terahertz quantum cascade lasers. N. J. Phys..

[27] Wang F (2015). Generating ultrafast pulses of light from quantum cascade lasers. Optica.

[28] Riesch, M. & Jirauschek, C. An open-source solver tool for the Maxwell-Bloch equations. https://github.com/mriesch-tum/mbsolve (2017).

[29] Riesch M, Tchipev N, Senninger S, Bungartz H-J, Jirauschek C (2018). Performance evaluation of numerical methods for the Maxwell–Liouville–von Neumann equations. Opt. Quantum Electron..

[30] Jirauschek C, Kubis T (2014). Modeling techniques for quantum cascade lasers. Appl. Phys. Rev..

[31] Jirauschek C, Riesch M, Tzenov P (2019). Optoelectronic device simulations based on macroscopic Maxwell–Bloch equations. Adv. Theory Simul..

[32] St-Jean MR (2017). Mode stabilization in quantum cascade lasers via an intra-cavity cascaded nonlinearity. Opt. Express.

[33] Piccardo M (2019). Radio frequency transmitter based on a laser frequency comb. Proc. Natl Acad. Sci. USA.

[34] Madeo J (2010). Frequency tunable terahertz interdigitated photoconductive antennas. Electron. Lett..

