# Supplementary material for: Ultrafast response of harmonic modelocked THz lasers
Source: Light Sci Appl. 2020 Apr 1;9:51. doi: 10.1038/s41377-020-0288-x (PMC7113272; doi:10.1038/s41377-020-0288-x)
Supplement: Supplementary file 1 — Supplementary Material [file 41377_2020_288_MOESM1_ESM.docx]

**Supplementary Material:**

**Ultrafast Response of Harmonic Modelocked THz Laser**

Feihu Wang,^1^ Valentino Pistore,^1^ Michael Riesch,^2^  Hanond Nong,^1^ Pierre-Baptiste Vigneron,^3^ Raffaele Colombelli,^3^ Olivier Parillaud,^4^ Juliette Mangeney,^1^ Jerome Tignon^1^, Christian Jirauschek^2^, and Sukhdeep S. Dhillon^1*^

*^1^Laboratoire de Physique de l’Ecole Normale Supérieure, ENS, Université PSL, CNRS, Sorbonne Université, Université de Paris, Paris, France*

*^2^ Department of Electrical and Computer Engineering, Technical University of Munich, Arcisstr. 21, 80333 Munich, Germany*

*^3^ Centre de Nanosciences et de Nanotechnologies, CNRS, Univ. Paris-Sud, Université Paris-Saclay, C2N-Orsay, 91405 Orsay Cedex, France*

*^4^ III-V lab, 1 avenue Augustin Fresnel, 91767 Palaiseau, France*

* Corresponding author - sukhdeep.dhillon@ens.fr

**Methods**

**QCL electrical and optical characteristics:**

The light-current-voltage characteristics are shown in Fig. S1 for a 6 mm long device at 10 K.

|  |
| --- |
| **Figure S1.** Light-current-voltage characteristics of the THz QCL operating at 2.45 THz at 10 K. |

**Ultrafast detection of the QCL emission:**

**Injection seeding**: The pulse characterization of the THz quantum cascade laser (QCL) is based on coherent sampling of the electric(E)-field using electro-optic detection. This technique requires to phase lock the emission of the THz QCL to a THz pulse, which in turn is locked to the repetition rate of a femtosecond laser. To fulfil this requirement, an established ultrafast injection seeding technique is employed. A broad-band THz pulse (seed) with a fixed phase is generated using a photoconductive switch excited by a 100 fs near-infrared pulse from a Ti: Sapphire laser. The THz seed pulse is injected into one end cavity of the QCL waveguide prior to gain switching the QCL with an electrical radio frequency (RF) pulse with a duration of a few nanoseconds. This allows the THz input pulse to be amplified and eventually seed the QCL emission, instead of being initiated by the QCL’s inherent spontaneous emission. Finally, a purge box with dry air is used to prevent absorption of the THz emission by atmospheric water.

**Modelocking**: To initiate the mode-locking regime, a microwave modulation of the QCL bias is applied close to the THz cavity round-trip frequency. The gain is modulated close to the QCL threshold. The microwave modulation is generated from the photo-excitation of an ultrafast photodiode by a pick-off beam of the Ti:Sapphire laser. The generated electrical signal consists of a comb of frequencies extending to ~20 GHz separated by the Ti:Sapphire repetition rate (76 MHz). A yttrium iron garnet bandpass filter is used to pick out a harmonic of the reference laser repetition rate close to the QCL cavity round-trip frequency, which is then amplified by a set of microwave power amplifiers. The proper synchronization of the THz seed pulse with the modulated QCL bias allows the detection of the QCL E-field as function of the time via electro-optic sampling. Further details of the technique can be found in Ref.  [2] and  [3].

**Modelling of QCL ultrafast response:**

To understand the measured harmonic time and spectral behaviour using simulation tools, we set up a model of the quantum cascade laser structure that consists of different gain and absorber regions along the propagation direction. The different regions aim to capture the influence of the microwave beatnote field on the active region. We found that the setup consisting of a 0.75 mm absorber, 1.5 mm gain, 1.5 mm absorber, 1.5 mm gain, and 0.75 mm absorber captures two characteristics of the measured result, namely the spacing of the different modes (equals 2*f_RT_*) and the distance between the two frequency lobes (15^th^ harmonic).

Both region types (i.e., gain and absorber) share the same electromagnetic properties, namely the refractive index *n*, the linear power loss *α*, and the overlap factor *Γ*. Additionally, the doping density *N* is the same in both regions. The quantum mechanical model of the electron dynamics bases on a three-level density matrix description, where the *ρ_11_* represents the population of the miniband and *ρ_22_* and *ρ_33_* are the populations of upper and lower lasing level, respectively. Between the latter two the energy difference corresponds to the transition frequency *f*. This lasing transition is further described by the dipole moment *d* and the dephasing time *T_deph_*. Finally, the non-radiative scattering between the energy levels is included using three scattering rates which correspond to the superlattice relaxation time as well as the lower and the upper lasing level lifetime, respectively.

| **Quantity** | **Symbol** | **Value (Gain)** | **Value (Loss)** | **Unit** | **Source** |
| --- | --- | --- | --- | --- | --- |
| **Refractive index** | ***n*** | 3.6 | 3.6 |  | * |
| **Linear Power Loss** | ***α*** | 16 | 16 | cm^-1^ | § |
| **Overlap Factor** | ***Γ*** | 1.0 | 1.0 |  | * |
| **Doping Density** | ***N*** | 5.585e+15 | 5.585e+15 | cm^-3^ | * |
| **Transition Frequency** | ***f*** | 2.415 | 2.415 | THz | # |
| **Dipole Moment** | ***d*** | 4.4 | 4.4 | nm | # |
| **Dephasing time** | ***T_deph_*** | 600 | 600 | fs | § |
| **Superlattice time** | ***τ_12_*** | 30 | 30 | ps | § |
| **Lower level lifetime** | ***τ_31_*** | 3 | 3 | ps | § |
| **Upper laser lifetime** | ***τ_23_*** | 10 | 2 | ps | § |

Table 1: Parameters of the numerical simulation. The source entry indicates how the parameter in question was determined: the asterisk * denotes known material parameters, the hash symbol # marks values determined by simulation, and the values chosen in the scope of parameter exploration are marked with § (range of reasonable parameter values from literature [1, 5, 6]).

This model was implemented using the mbsolve simulation tool [4]. We determined the unknown material parameter values by either simulation or by using reasonable values from related literature [1, 5, 6]. Then, we assumed that the gain and loss regions mainly differ in the upper lasing level life time, which determines whether the region acts as gain or as absorber medium, and varied the length of the loss region in order to find parameter combinations for which second harmonic behaviour occurs. We found that a possible combination is the parameter set listed in Tab. 1.

The simulation source code is publicly available in our GitHub repository [4]. The simulation script in our data repository (https://doi.org/10.5281/zenodo.3689614) may serve as starting point for the user. It should be noted that due to random initialization, the amplitudes of the modes may vary between simulation runs. The qualitative statements, however, remain the same.

|  |
| --- |
| Figure S2. a) Schematic of QCL with gain-loss-gain regions (GAG). Maxwell-Bloch simulations showing b) time response for GAG geometry; c) spectral response without loss and d) spectral response with GAG geometry. |

Figure S2a shows a schematic of a modulation at the second harmonic resulting in a gain-loss-gain system, in contrast to that shown in the main manuscript that simulated extra absorption regions. Here, the calculated time response (depicted in Fig. S2b) shows a modulation at the second harmonic but multiple pulse behaviour per round trip is not observed. Although the calculated spectral response shows QCL modes separated by the second harmonic (Fig. S2d), compared to the case of zero loss regions that operates at the fundamental frequency (Fig. S2c), the different frequency bands separated by 100 GHz measured in the experiments cannot be observed.

[1] M. I. Amanti, G. Scalari, R. Terazzi, M. Fischer, M. Beck, J. Faist, A. Rudra, P. Gallo, and E. Kapon, New J. Phys. **11**, 125022 (2009).

[2] D. Oustinov, N. Jukam, R. Rungsawang, J. Madéo, S. Barbieri, P. Filloux, C. Sirtori, X. Marcadet, J. Tignon, and S. Dhillon, Nat. Commun. **1**, 1 (2010).

[3] J. R. Freeman, J. Maysonnave, H. E. Beere, D. A. Ritchie, J. Tignon, and S. S. Dhillon, Opt. Express **21**, 16162 (2013).

[4] M. Riesch and C. Jirauschek, https://github.com/mriesch-tum/mbsolve (2017).

[5] P. Tzenov, I. Babushkin, R. Arkhipov, M. Arkhipov, N. Rosanov, U. Morgner, and C. Jirauschek, New J. Phys. **20**, 053055 (2018).

[6] P. Tzenov, D. Burghoff, Q. Hu, and C. Jirauschek, Opt. Express **24**, 23232 (2016).
